# Comprehensive analysis of PHF5A as a potential prognostic biomarker and therapeutic target across cancers and in hepatocellular carcinoma

**DOI:** 10.1186/s12885-024-12620-z

**Published:** 2024-07-19

**Authors:** Qianqian Cheng, Wenbin Ji, Zhenyu Lv, Wei Wang, Zhaiyue Xu, Shaohua Chen, Wenting Zhang, Yu Shao, Jing Liu, Yan Yang

**Affiliations:** 1 Department of Medical Oncology, The First Affiliated Hospital of Bengbu Medical University, 233004 Bengbu, China; 2 Department of Gastroenterology, The Third People’s Hospital of Bengbu, 233004 Bengbu, China; 3 School of Medical, Southeast University, 210000 Nanjing, China; 4 Department of Pathology, The First Affiliated Hospital of Bengbu Medical University, 233004 Bengbu, China; 5 National Drug Clinical Trial Center, The First Affiliated Hospital of Bengbu Medical University, 233004 Bengbu, China

**Keywords:** Angiogenesis, Hepatocellular carcinoma, Pan-cancer, Prognosis, Treatment response, Tumor immunity

## Abstract

**Objective:**

Cancer is a predominant cause of death globally. PHD-finger domain protein 5 A (PHF5A) has been reported to participate in various cancers; however, there has been no pan-cancer analysis of PHF5A. This study aims to present a novel prognostic biomarker and therapeutic target for cancer treatment.

**Methods:**

This study explored PHF5A expression and its impact on prognosis, tumor mutation burden (TMB), microsatellite instability (MSI), functional status and tumor immunity across cancers using various public databases, and validated PHF5A expression and its correlation with survival, immune evasion, angiogenesis, and treatment response in hepatocellular carcinoma (HCC) using bioinformatics tools, qRT-PCR and immunohistochemistry (IHC).

**Results:**

PHF5A was differentially expressed between tumor and corresponding normal tissues and was correlated with prognosis in diverse cancers. Its expression was also associated with TMB, MSI, functional status, tumor microenvironment, immune infiltration, immune checkpoint genes and tumor immune dysfunction and exclusion (TIDE) score in diverse malignancies. In HCC, PHF5A was confirmed to be upregulated by qRT-PCR and IHC, and elevated PHF5A expression may promote immune evasion and angiogenesis in HCC. Additionally, multiple canonical pathways were revealed to be involved in the biological activity of PHF5A in HCC. Moreover, immunotherapy and transcatheter arterial chemoembolization (TACE) worked better in the low PHF5A expression group, while sorafenib, chemotherapy and AKT inhibitor were more effective in the high expression group.

**Conclusions:**

This study provides a comprehensive understanding of the biological function of PHF5A in the carcinogenesis and progression of various cancers. PHF5A could serve as a tumor biomarker related to prognosis across cancers, especially HCC, and shed new light on the development of novel therapeutic targets.

**Supplementary Information:**

The online version contains supplementary material available at 10.1186/s12885-024-12620-z.

## Introduction

Cancer incidence and mortality rates are quickly increasing, posing a global public health challenge [1–3]. In the past several years, genetic testing technology has made its way into clinical practice. Identifying genes affecting tumor development and targeting abnormally mutated genes has become one of the most prevalent antitumor therapeutic approaches. Therefore, exploring carcinogenesis mechanisms and searching for biomarkers or therapeutic targets related to prognosis are urgent issues to be addressed.

PHD finger protein 5 A (PHF5A) has a wide distribution in eukaryotic nuclei and can engage in multiple biological events, including chromatin remodeling [4], DNA damage repair [5] and the growth and differentiation of cells [6]. Several studies, including ours, have revealed that PHF5A can exert a regulatory function in the onset and progression of an array of malignancies, including endometrial cancer [7], lung cancer [8–10], breast cancer [11], colorectal cancer [12, 13], pancreatic cancer [14], gastric cancer [15] and head and neck squamous cell carcinoma [16], by mediating abnormal alternative splicing of target genes, regulating crucial signaling pathways, or acting as a proto-oncogene/protein, transcription factor or cofactor. Therefore, the relationship between PHF5A and tumors has emerged as a focal point in the oncology research field [17, 18]. For hepatocellular carcinoma (HCC), a previous study illustrated that elevated PHF5A expression facilitates invasion and migration via the NF-KB pathway [19], but comprehensive correlation analysis of PHF5A with tumor immunity and angiogenesis in HCC has not been performed. Most importantly, prior research has primarily examined the role of PHF5A in specific cancers, with no comprehensive research exploring survival significance or biological role of PHF5A across cancers.

Therefore, this research systematically explored PHF5A expression and its correlation with prognosis, tumor mutation burden (TMB), microsatellite instability (MSI), functional status and tumor immunity across cancers and validated PHF5A expression and its association with survival, immune evasion, angiogenesis, and treatment response in HCC, with the aim of presenting a novel prognostic biomarker and therapeutic target for cancer treatment.

## Materials and methods

### PHF5A expression across cancers

Differential expression of PHF5A mRNA and protein in tumor and matching normal tissues was evaluated utilizing the TIMER database [20] (accessed in May 2023) and UALCAN database [21] (accessed in May 2023), respectively. From the HPA database [22] (accessed in May 2023), immunohistochemistry (IHC) images of PHF5A in tumor and matching normal tissues were retrieved. The acronyms for the different types of cancer were presented in Supplementary Table 1.

### Prognostic role of PHF5A across cancers

The median PHF5A expression was utilized to categorize patients exhibiting high or low PHF5A expression levels. A Cox regression model was employed to assess the link of PHF5A expression with overall survival (OS), disease-specific survival (DSS), and progression-free interval (PFI), and these findings were visualized with forest plots. Cancers whose prognosis was affected by PHF5A expression level were screened via a Cox regression model, and Kaplan‒Meier curves for the OS, DSS and PFI of different PHF5A expression groups were plotted for these cancers, with survival analysis of patients with different PHF5A expression evaluated utilizing the Log-rank test. The above procedures were conducted at Xiantao Academic [23] (accessed in May 2023).

### PHF5A mutation landscape analysis across cancers

The cBioPortal website [24] (accessed in May 2023) was implemented to evaluate PHF5A mutation information, with the cohort “Pan-cancer analysis of whole genomes (ICGC/TCGA, Nature 2020)”. Spearman correlation analysis of PHF5A expression with TMB and MSI was performed using the Assistant for Clinical Bioinformatics (ACLBI) database [25] (accessed in July 2023).

### PHF5A analysis at the single-cell level with public databases

The link of PHF5A expression with functional status in diverse cancers was studied utilizing the CancerSEA database [26] (accessed in August 2023). The connection of PHF5A expression with the tumor microenvironment (TME) of LIHC, NSCLC, PAAD, STAD was evaluated at the single-cell sequencing level using the TISCH website [27] (accessed in May 2023).

### Association of PHF5A with immune prospects across cancers

Heatmaps of PHF5A expression with immune cell infiltration and key immune checkpoint genes across cancers were plotted using Spearman correlation analysis, and tumor immune dysfunction and exclusion (TIDE) scores of different PHF5A expression groups were calculated and compared, and the significance of two groups was tested by the Wilcoxon test. The above analyses were conducted via the ACLBI database (accessed in July 2023). The connection of PHF5A expression with immune score in various cancers was explored with the Sangerbox 3.0 platform [28] (accessed in July 2023), with Spearman correlation analysis utilized.

### qRT-PCR

Nanjing KeyGen Biotech. Co., Ltd. (Nanjing, China) and the Chinese Academy of Sciences (Shanghai, China) provided the normal hepatic cell line LO2 and HCC cell lines (Huh7, Hep3B, BEL-7404 and SMMC-7721), respectively. These cell lines were utilized for qRT‒PCR. PCR quantification operations were described in our earlier studies [29, 30], and Supplementary Table 2 provided the primers.

The data were analyzed after the above experiment was repeated three times. GAPDH was selected as the internal reference gene and the relative expression of PHF5A was calculated using the 2^−ΔΔCt^ (ΔCt = CT ^PHF5A^- CT ^GAPDH^) method.

### Human samples collection and patients follow-up

Forty-four pairs of tumor and adjacent non-tumor tissues from HCC patients who underwent surgery at our hospital between January 2017 and December 2019 were collected, and we further recorded their clinical data and followed up these HCC patients through the medical record system and telephone counseling. The cutoff date for the follow-up was June 1, 2023. Additionally, twenty pairs of human cholangiocarcinoma (CCA) and matched adjacent non-tumor tissue samples were also obtained at the time of surgery in the same period.

### Immunohistochemistry (IHC)

Tissue blocks were embedded, sectioned (4 μm), baked (75 °C, 6 h), and then the steps of IHC are based on dewaxing, gradient ethanol hydration, antigen repair, endogenous peroxidase blockade, serum blockade, antigen-antibody reaction, DAB chromatography, hematoxylin re-staining, gradient ethanol dehydration and neutral gum sealing, as described in our previous experiment procedures [30, 31]. Proteintech (Wuhan, China) supplied the anti-PHF5A, VEGFA, and VEGFR2 primary antibodies. The dilution ratio of anti-PHF5A antibody was 1:50, and the dilution ratio of anti-VEGFA and VEGFR2 antibodies was 1:200. Maishin Biotechnology (Fuzhou, China) provided anti-CD34 antibody and anti-CD3+/CD4+/CD8 + T-cell primary antibodies, and they were all read-to-use antibodies. All operating procedures strictly adhered to kit instructions. The staining results were blindly evaluated by two pathologists using an optical microscope. Microscopy showing tan, brownish-yellow or yellow granules in the nucleus [32], was considered to indicate PHF5A positivity.

### Clinicopathological data analysis of HCC patients in the study cohort

Clinicopathological information, including sex, age, hepatitis, AFP, Child‒Pugh, tumor size, tumor number, BCLC stage and postoperative therapy, of HCC patients was recorded and analyzed by stratification by PHF5A expression level, and the χ^2^ test or Fisher’s exact test was performed where appropriate.

### Relationship of PHF5A with immune cell infiltration in HCC

The association of PHF5A with immune cell infiltration across cancers, including HCC, was mentioned as above in the section of association of PHF5A with immune prospects across cancers. To further confirm the connection between PHF5A and immune cell distribution, samples positive and negative for PHF5A expression were chosen for CD3+/CD4+/CD8 + T-cell IHC staining, and experimental information was described in the section of immunohistochemistry (IHC).

### Gene set enrichment analysis (GSEA)

HCC transcriptome information was obtained from the TCGA database [33] (accessed in May 2023). Input files were acquired and analyzed for gene set enrichment via Perl software and GSEA 4.3.2. | Normalized enrichment score (NES) |>1, NOM p value < 0.05 and FDR q-value < 0.25 were defined as notably enriched [34].

### Correlation between PHF5A expression and angiogenesis in HCC

The expression correlation of PHF5A with VEGFA, VEGFR2 and CD34 was analyzed using the GEPIA website [35] (accessed in May 2023). To verify the relationship between PHF5A expression and angiogenesis, samples positive and negative for PHF5A expression were selected for VEGFA, VEGFR2 and CD34 IHC staining, and experimental information was described in the section of immunohistochemistry (IHC).

### Correlation between PHF5A and treatment response in HCC

Immune phenotype score (IPS) data of TCGA-LIHC specimens were downloaded via the TCIA website [36] (accessed in July 2023), and immunotherapy efficacy was assessed by IPS. PHF5A expression and treatment information of transcatheter arterial chemoembolization (TACE) were obtained using the Gene Expression Omnibus (GEO) database [37] (GSE104580, accessed in July 2023) for 147 HCC patients. The median inhibitory concentration (IC50) for sorafenib, doxorubicin, 5-fluorouracil, gemcitabine and AKT inhibitor were calculated in HCC using the R package “pRRophetic”, and significant differences of two PHF5A expression groups were compared through the Wilcoxon test. Spearman correlation analysis was utilized to evaluate the association of IC50 and PHF5A expression.

### Other statistical methods

R software (4.3.2), Perl software, GraphPad Prism 8.0 and SPSS 25.0. The t test or Wilcoxon test was appropriate for two-group comparisons, and differences among three groups were assessed with the Kruskal‒Wallis test. The χ^2^ test or Fisher’s exact test was utilized to compare percentages. Survival analysis was performed via the log-rank test and Cox regression model. Statistical significance at *P* < 0.05.

## Results

### PHF5A expression across cancers

**Fig. 1:**
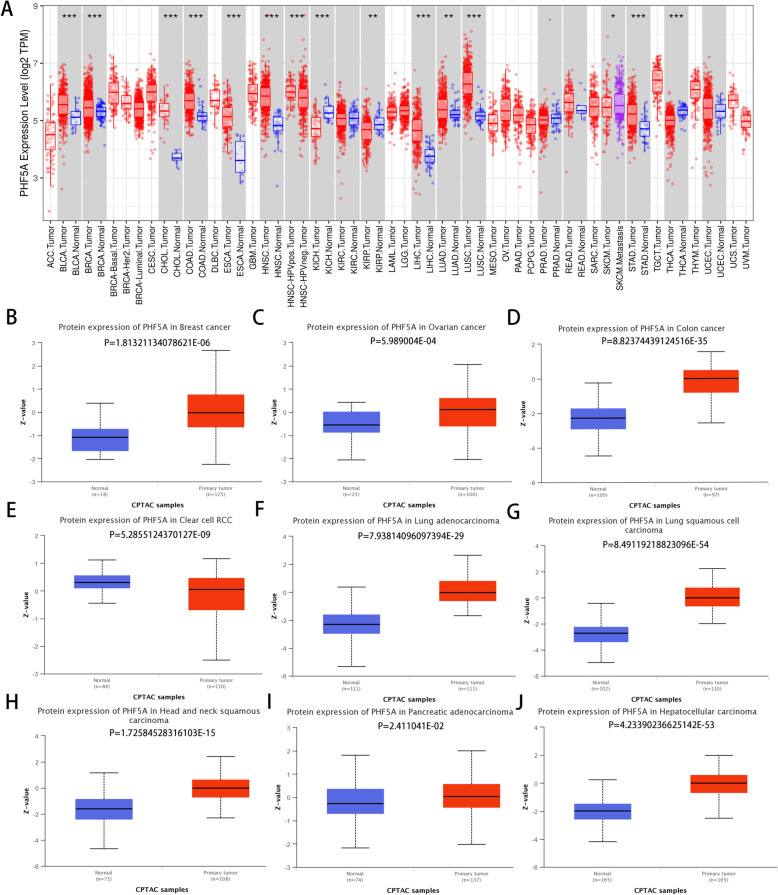
PHF5A expression across cancers in public databases. **A** PHF5A mRNA expression across cancers according to the TIMER database. **B**-**J** PHF5A protein expression in BRCA, OV, COAD, KIRC, LUAD, LUSC, HNSC, PAAD and HCC based on the UALCAN website. *P < 0.05, **P < 0.01, ***P < 0.001

According to the TIMER database, when compared with that in matching normal tissues, PHF5A mRNA expression was considerably elevated in tumor tissues, including BLCA, BRCA, CHOL, COAD, ESCA, HNSC, LIHC, LUAD, LUSC and STAD tissues, but was significantly reduced in KICH, KIRP, and THCA tissues (all *P* < 0.05, Fig. 1A). Figure 1B-J demonstrated that PHF5A protein expression was substantially elevated in BRCA, OV, COAD, LUAD, LUSC, HNSC, PAAD and HCC tissues but decreased in KIRC tissues (all *P* < 0.05). IHC images of PHF5A in various tumor and matching normal tissues were illustrated in Supplementary Fig. 1.

### Prognostic significance of PHF5A across cancers

**Fig. 2:**
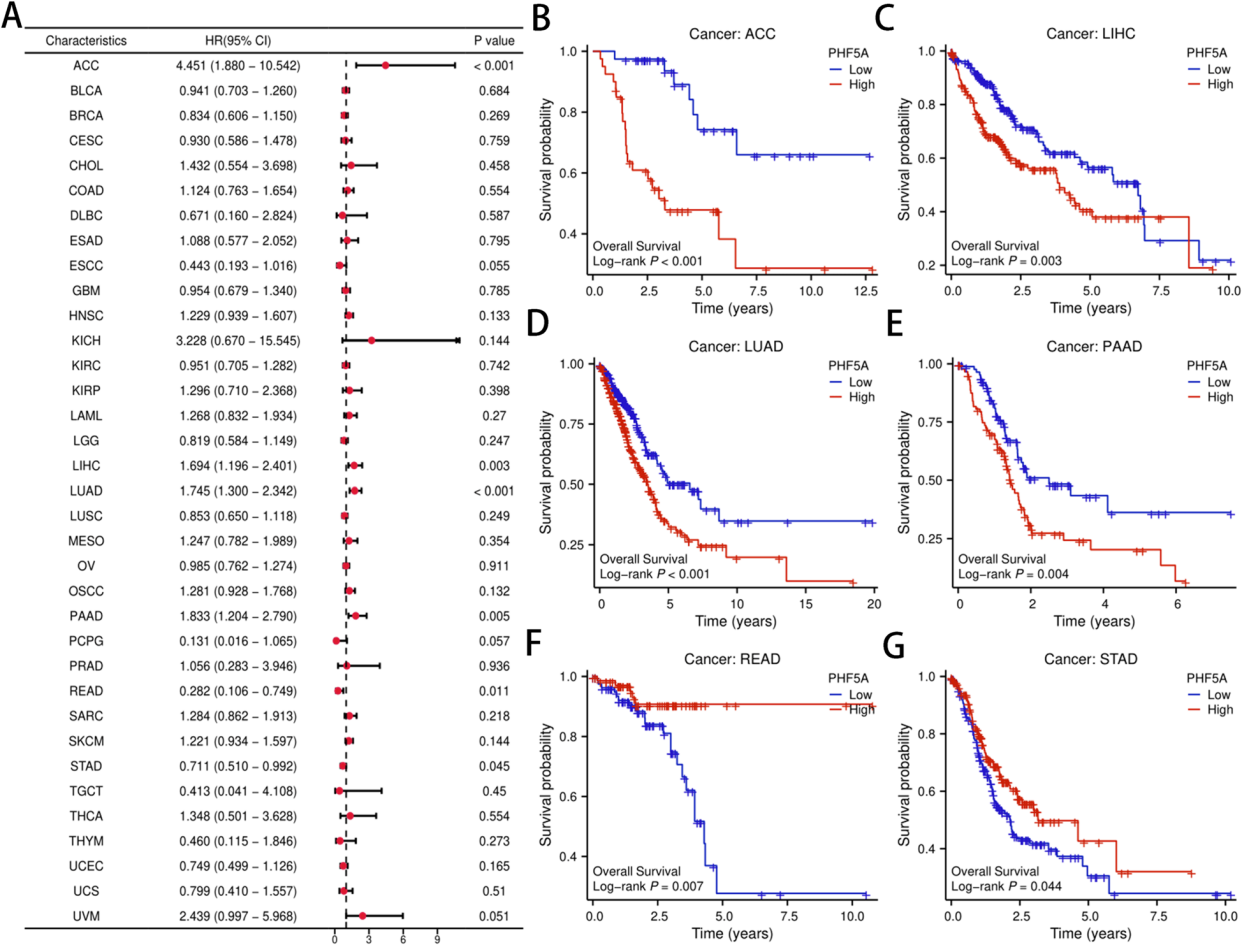
Correlation of PHF5A with overall survival (OS) across cancers in TCGA database. **A** Cox regression model of PHF5A for OS across cancers. **B**-**G** Kaplan‒Meier curves for OS of different PHF5A expression groups in ACC, LIHC, LUAD, PAAD, READ and STAD

PHF5A expression was adversely linked with patient OS in ACC, LIHC, LUAD and PAAD, with DSS in ACC, LIHC, LUAD and PAAD, and with PFI in ACC, KIRP, LIHC, PAAD, PRAD and UVM. However, PHF5A expression had a favorable connection with patient OS in READ and STAD, with DSS in STAD, and with PFI in READ, STAD and UCEC (all *P* < 0.05, Fig. 2 and Supplementary Fig. 2).

### Mutation landscape of PHF5A across cancers

**Fig. 3:**
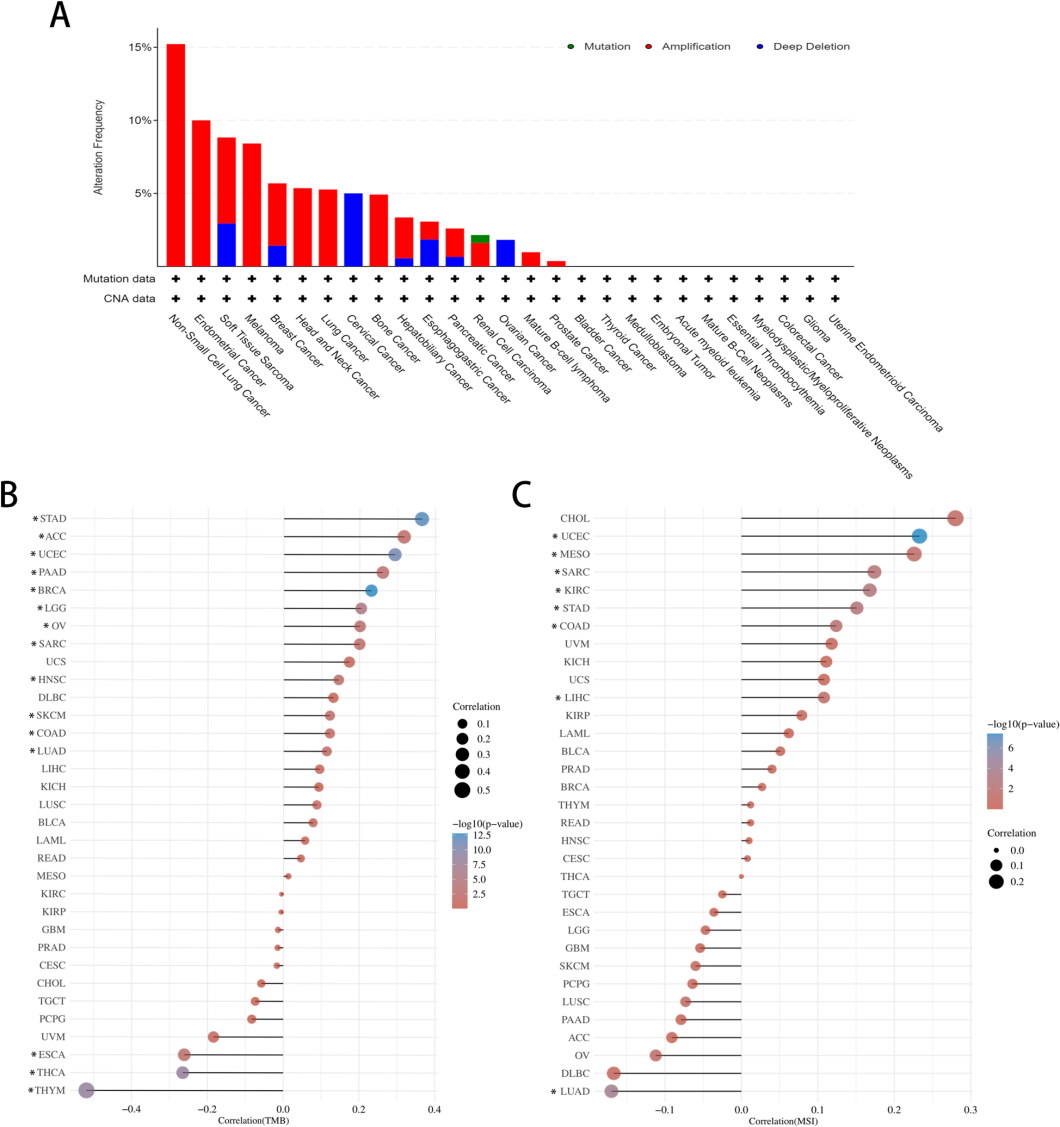
PHF5A mutation landscape analysis across cancers. **A** PHF5A mutation across cancers from the cBioPortal website. **B**, **C** Association of PHF5A expression with TMB and MSI. *P < 0.05

Figure 3A indicated that the main form of alteration in the PHF5A gene was amplification, and the top 5 cancers in terms of PHF5A mutation frequency were non-small cell lung cancer (15.22%), endometrial cancer (10.00%), soft tissue sarcoma (8.82%), melanoma (8.41%) and breast cancer (5.69%). PHF5A expression was favorably connected to TMB status in ACC, BRCA, COAD, HNSC, LGG, LUAD, OV, PAAD, SARC, SKCM, STAD and UCEC, and to MSI in COAD, KIRC, LIHC, MESO, SARC, STAD and UCEC, but had a negative association with TMB in ESCA, THCA and THYM, and with MSI in LUAD (all *P* < 0.05, Fig. 3B, C).

### Single-cell level analysis of PHF5A with public databases

**Fig. 4:**
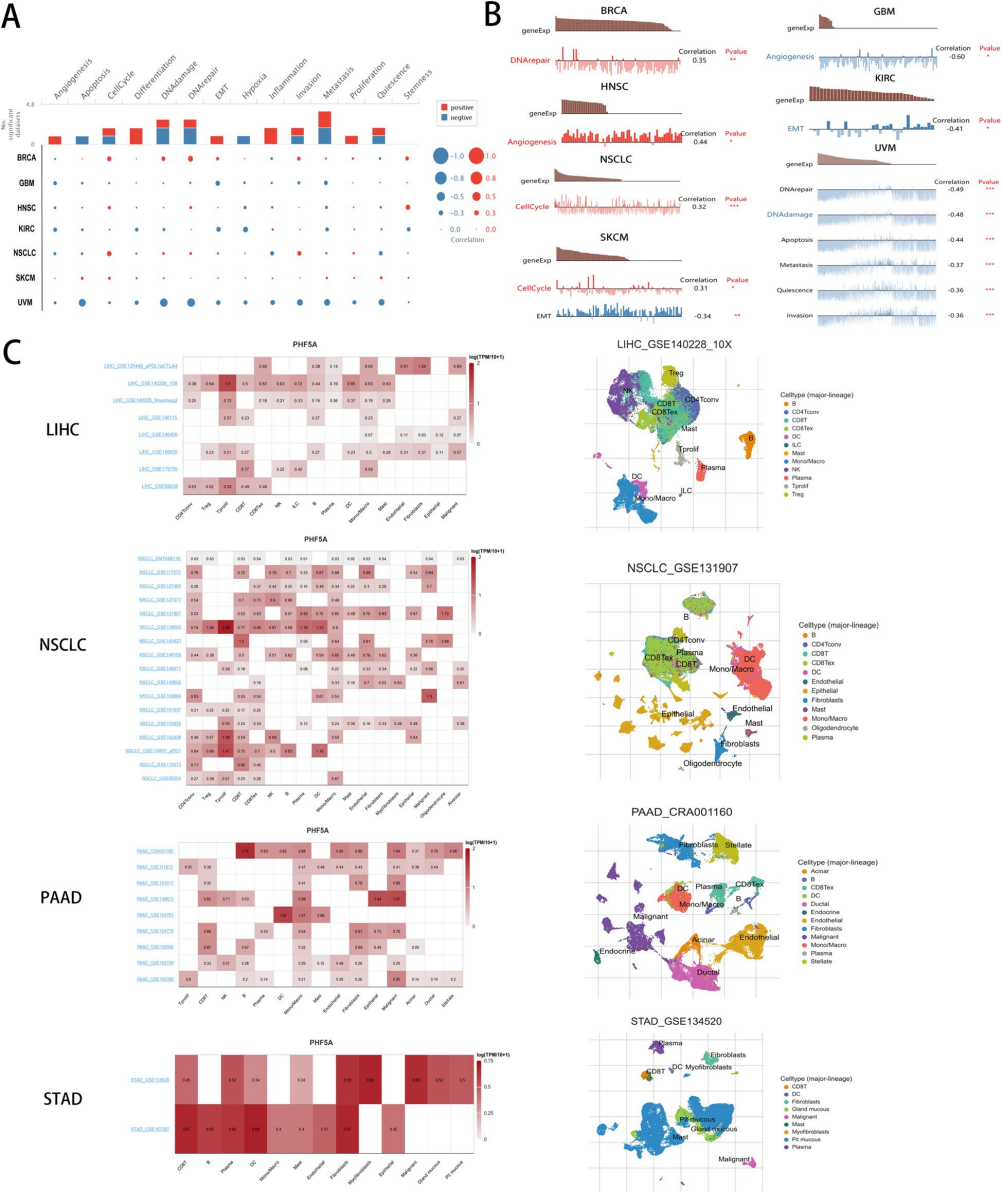
Single-cell level analysis of PHF5A with public databases. **A** Correlation of PHF5A expression with functional status in diverse cancers with the CancerSEA database. **B** Functional association of PHF5A in BRCA, GBM, HNSC, KIRC, NSCLC, SKCM and UVM from the CancerSEA database. **C** Correlation of PHF5A with the TME of LIHC, NSCLC, PAAD and STAD on the TISCH website

PHF5A expression was related to diverse functional statuses in various malignancies (Fig. 4A). PHF5A expression had a favorable connection with DNA repair in BRCA, angiogenesis in HNSC, and cell cycle in NSCLC and SKCM, but was inversely related to angiogenesis in GBM, EMT in KIRC and SKCM, and DNA repair, DNA damage, apoptosis, metastasis, quiescence, and invasion in UVM (Fig. 4B). Considering that PHF5A expression can affect OS of ACC, LIHC, LUAD, PAAD, READ and STAD and that TISCH online tool lacks datasets for ACC and READ, this study analyzed the association of PHF5A with TME in LIHC, NSCLC, PAAD and STAD at the single-cell sequencing level, which indicated that PHF5A was relevant to the TME in LIHC, NSCLC, PAAD and STAD and could be detected in malignant cells, immune cells and other cells (Fig. 4C).

### Correlation between PHF5A and tumor immunity

**Fig. 5:**
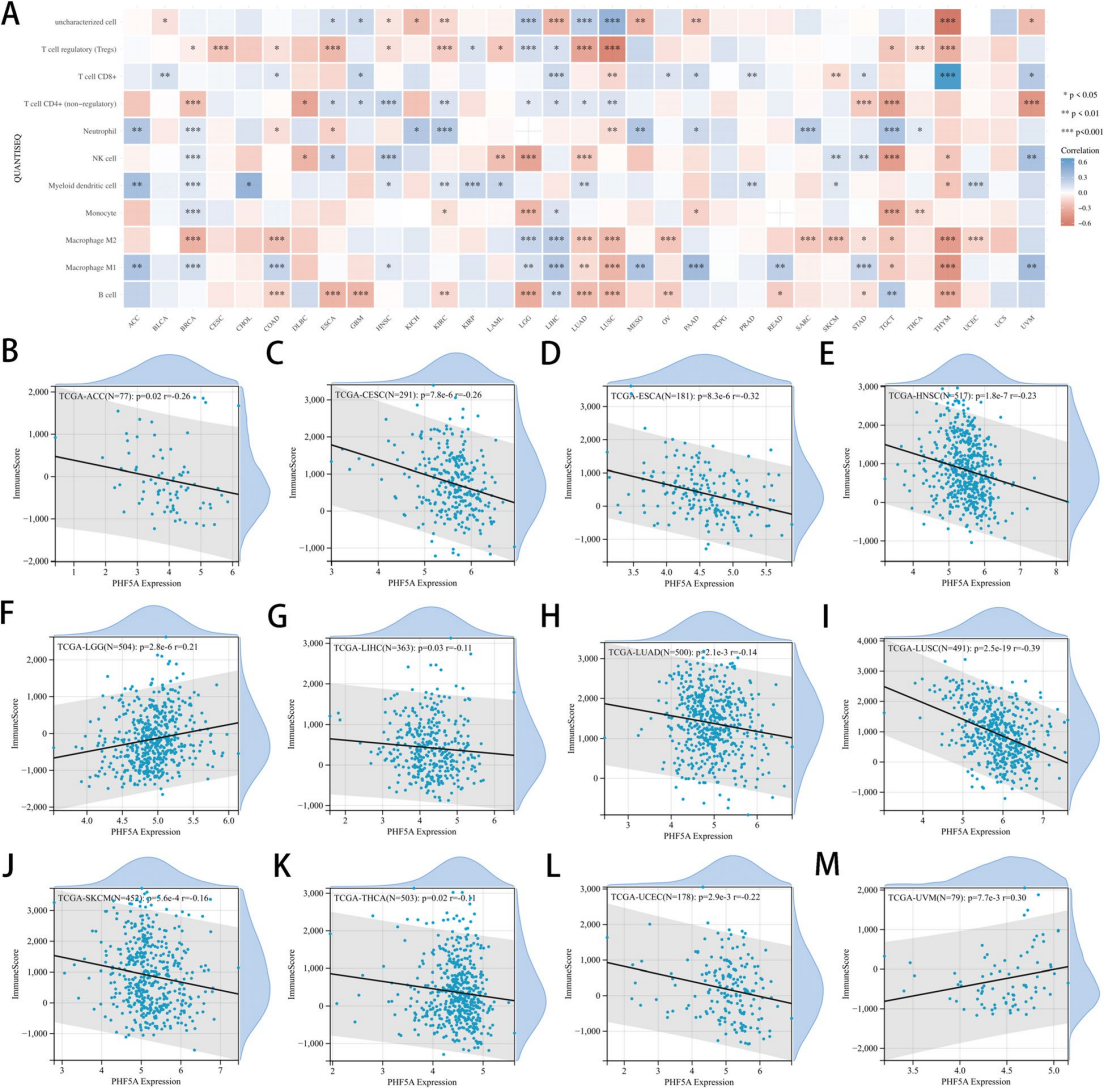
Association of PHF5A expression with immune cell infiltration. **A** Connection of PHF5A expression with immune cell infiltration across cancers (QUANTISEQ algorithm). **B**-**M** Relation of PHF5A expression with immune score in ACC, CESC, ESCA, HNSC, LGG, LIHC, LUAD, LUSC, SKCM, THCA, UCEC and UVM. *P < 0.05, **P < 0.01, ***P < 0.001

PHF5A expression was strongly connected to immune cell infiltration in various malignancies (Fig. 5A). Additionally, PHF5A expression was adversely correlated with immune scores of ACC, CESC, ESCA, HNSC, LIHC, LUAD, LUSC, SKCM, THCA and UCEC, while PHF5A expression was favorably related to immune scores of LGG and UVM (all *P* < 0.05, Fig. 5B-M).

**Fig. 6:**
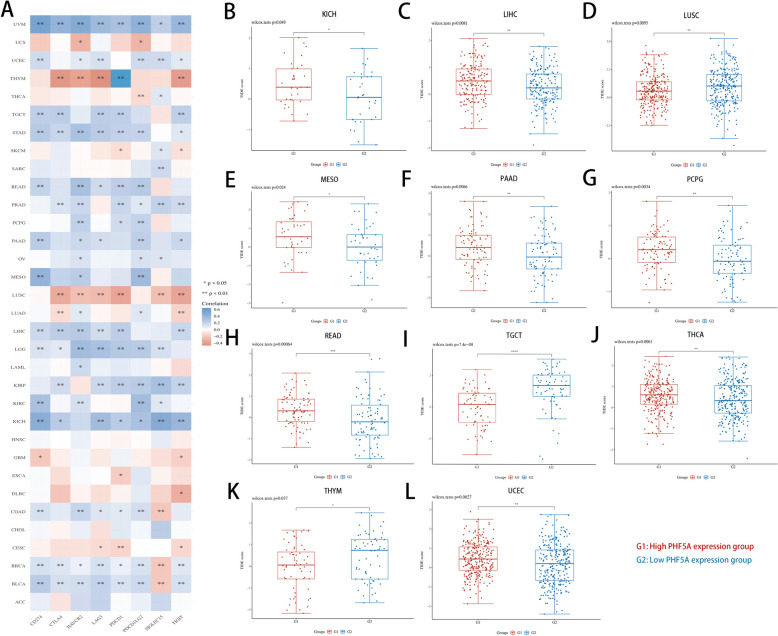
Value of PHF5A in predicting immunotherapy response with the ACLBI database. **A** Expression correlation of PHF5A with immune checkpoint genes. **B**-**L** Differences in TIDE scores of different PHF5A expression groups with KICH, LIHC, LUSC, MESO, PAAD, PCPG, READ, TGCT, THCA, THYM and UCEC. *P < 0.05, **P < 0.01, ***P < 0.001

PHF5A was closely correlated with immune checkpoint gene expression in diverse malignancies (Fig. 6A). The high PHF5A expression group exhibited elevated TIDE scores in KICH, LIHC, MESO, PAAD, PCPG, READ, THCA and UCEC, whereas the high PHF5A expression group had lower TIDE scores in LUSC, TGCT and THYM (all *P* < 0.05, Fig. 6B-L).

### PHF5A expression validation and its effect on prognosis in HCC

**Fig. 7:**
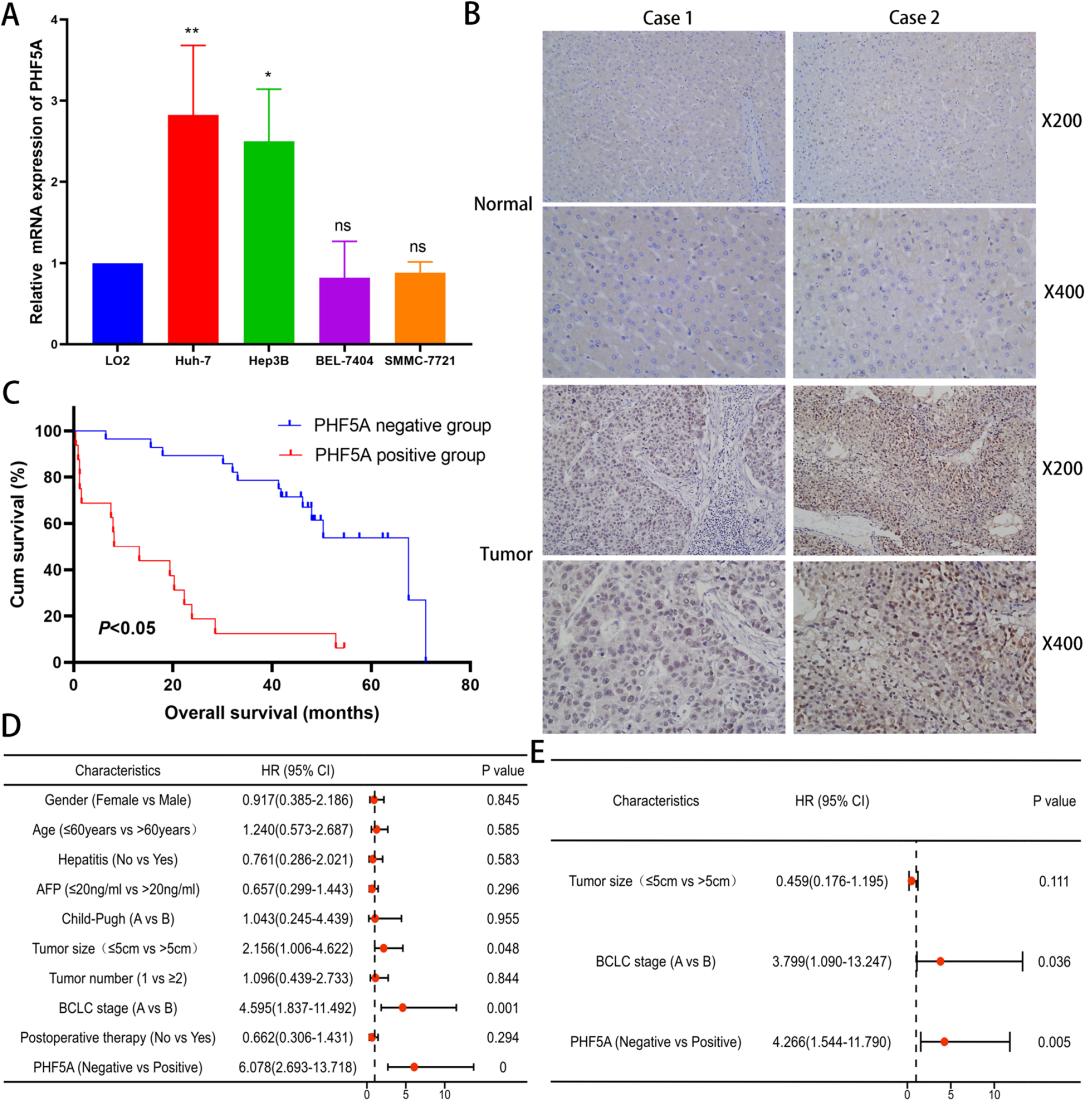
PHF5A expression validation and its effect on prognosis in HCC in the study cohort. **A** Relative mRNA expression of PHF5A in HCC cell lines (Huh-7, Hep3B, BEL-7404 and SMMC-7721) compared to that in the normal hepatic cell line LO2 (*N* = 3). **B** Representative images of PHF5A expression in normal liver and HCC tissues using IHC. **C** Kaplan‒Meier curves for the OS of different PHF5A expression groups in HCC patients (*N* = 44). **D**, **E** Univariate and multivariate Cox regression models of clinicopathological characteristics in HCC (*N* = 44). *P < 0.05, **P < 0.01, ns: not significant

qRT-PCR revealed that compared to that in LO2 cells, PHF5A expression was 1.82-fold higher in Huh-7 cells and 1.50-fold higher in Hep3B cells (both *P* < 0.05, Fig. 7A). PHF5A was situated mainly in the nucleus and demonstrated to be upregulation in HCC tissues via IHC (Fig. 7B). The expression model of PHF5A was also true in CCA when we expanded experimental validation to other cancer types (Supplementary Fig. 3).

The HCC study cohort consisted of 44 surgical samples, of which 28 HCC samples were negative for PHF5A expression and 16 HCC samples were positive for PHF5A expression, with a positive expression rate of PHF5A of 36.36% and a median survival time of 41.8 months. PHF5A expression exhibited a close link to tumor size and BCLC stage (both *P* < 0.05, Supplementary Table 3). Figure 7C revealed the inverse connection of PHF5A expression with prognosis (*P* < 0.05). Cox regression model has indicated that PHF5A expression and BCLC stage could independently influence OS in HCC (both *P* < 0.05, Fig. 7D, E).

### Relationship of PHF5A expression with immune cell distribution in HCC

**Fig. 8:**
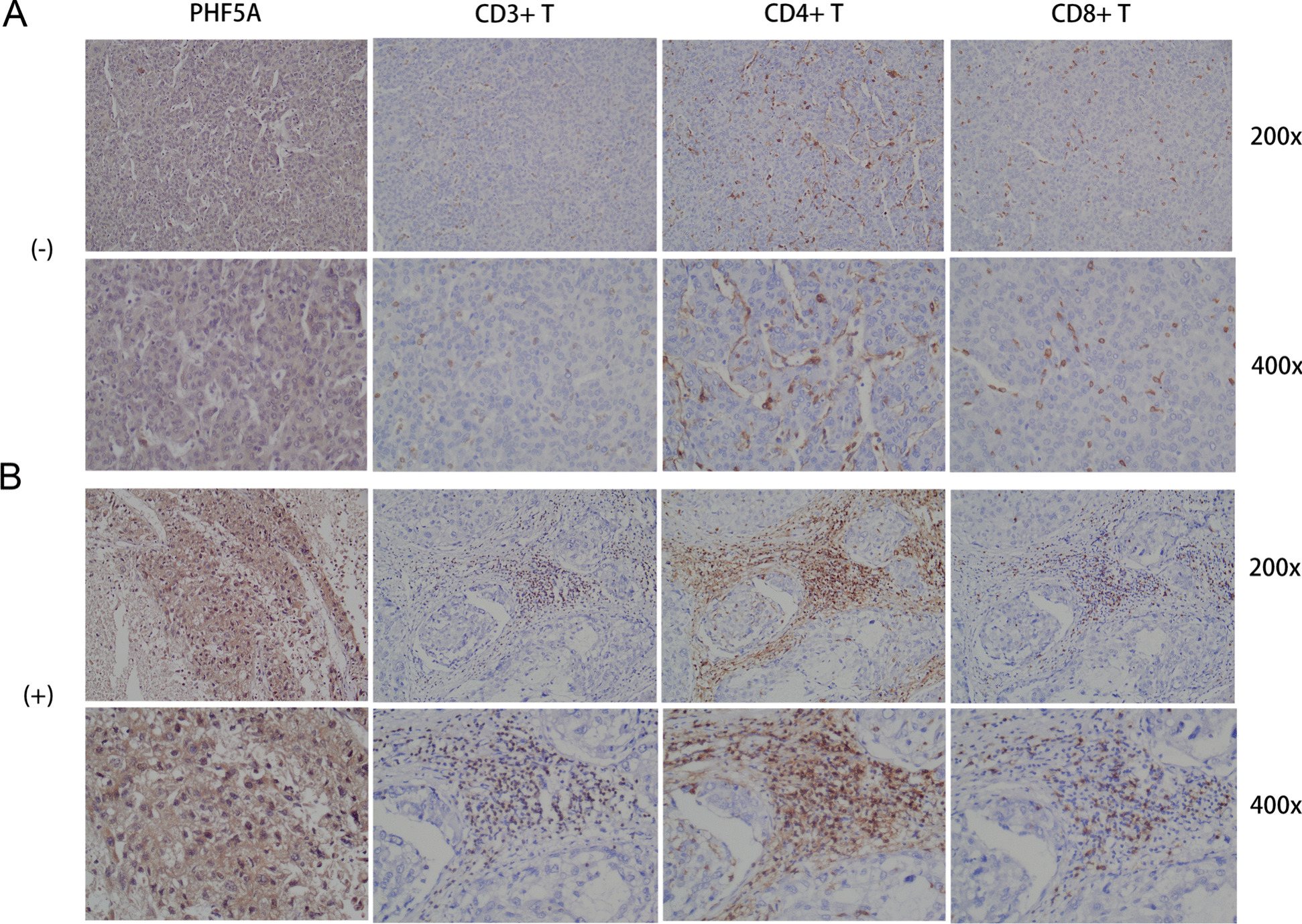
Immune cell infiltration at different PHF5A expression levels. **A** CD3+, CD4+, and CD8 + T-cell infiltration in the PHF5A-negative group. **B** CD3+, CD4+, and CD8 + T-cell infiltration in the PHF5A-positive group

In the study cohort, CD3+, CD4+, and CD8 + T cells were evenly scattered in the HCC tumor parenchyma in samples that were negative for PHF5A expression (Fig. 8A), whereas in samples that were positive for PHF5A expression, CD3+, CD4+, and CD8 + T cells were concentrated in the tumor periphery and rarely infiltrated the tumor parenchyma (Fig. 8B).

### GSEA of PHF5A in HCC

**Fig. 9:**
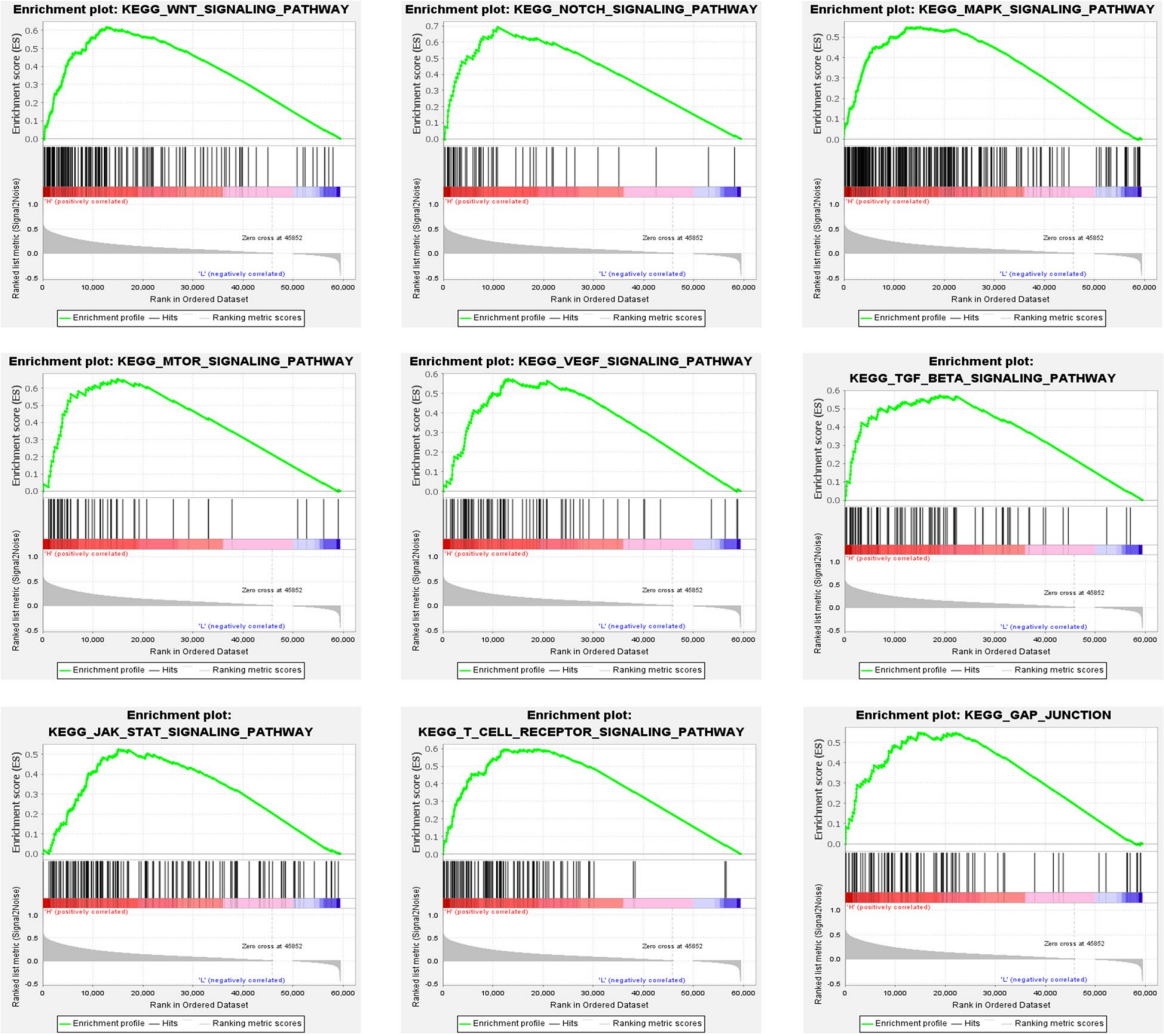
GSEA pathways activated by up-regulation of PHF5A in HCC

GSEA revealed that PHF5A could possibly engage in HCC development via the Wnt signaling pathway, Notch signaling pathway, MAPK signaling pathway, mTOR signaling pathway, VEGF signaling pathway, TGF-β signaling pathway, JAK STAT signaling pathway, T-cell receptor signaling pathway and gap junction (Fig. 9 and Supplementary Table 4).

### Association between PHF5A and angiogenesis in HCC

**Fig. 10:**
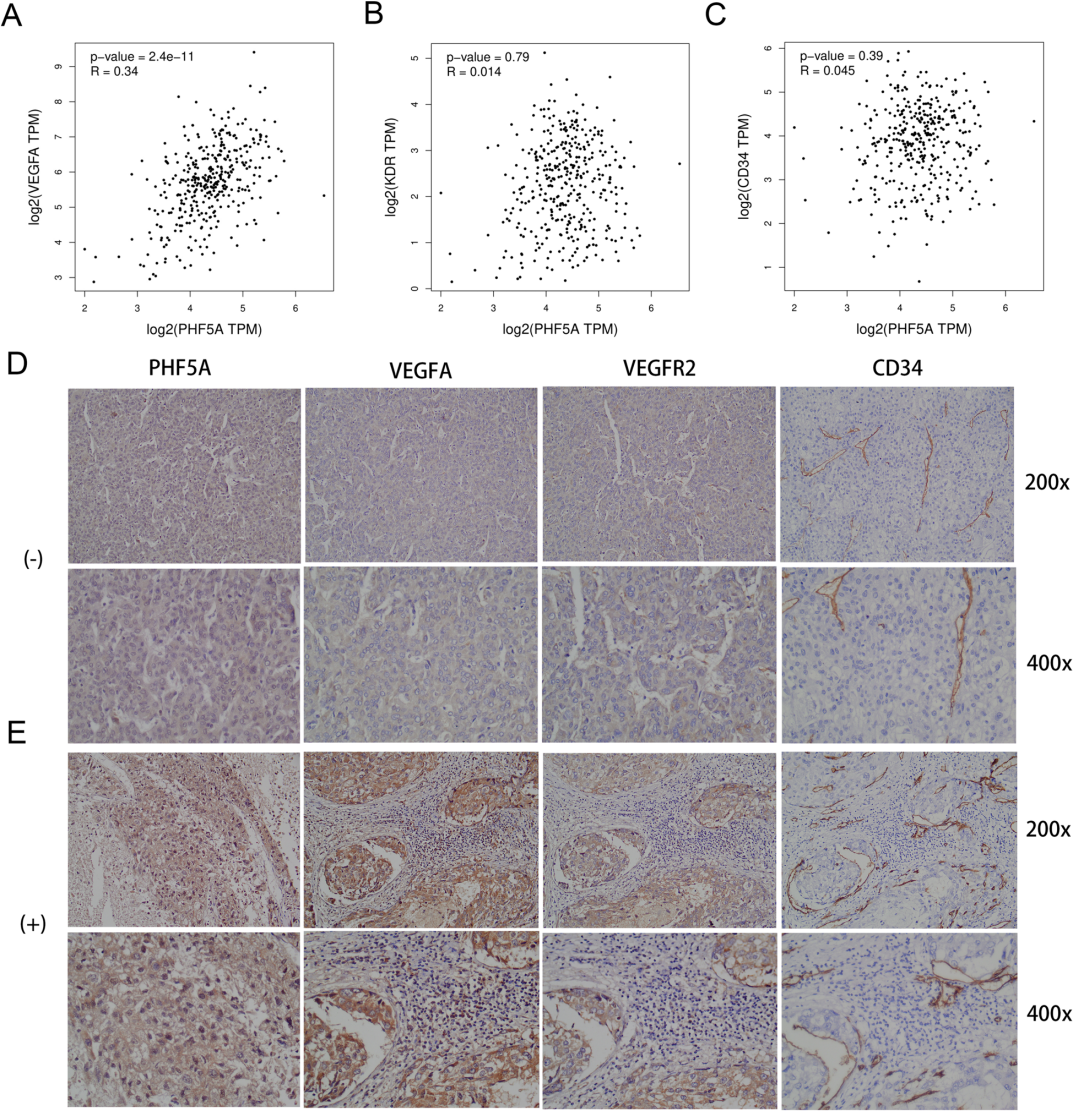
Correlation of PHF5A expression and angiogenesis in HCC. **A**-**C** Expression relationship of PHF5A with VEGFA, VEGFR2 and CD34 in the GEPIA database. **D** VEGFA, VEGFR2 and CD34 expression in the PHF5A-negative group. **E** VEGFA, VEGFR2 and CD34 expression in the PHF5A-positive group

Due to the well-known role of angiogenesis in HCC and the preliminary finding that the VEGF signaling pathway is implicated in PHF5A activity, relationship of PHF5A with angiogenesis was further explored. GEPIA database demonstrated that PHF5A expression was favorably linked with VEGFA expression but not with VEGFR2 or CD34 expression (Fig. 10A-C). According to the findings in the study cohort, PHF5A expression might be positively correlated with VEGFA, VEGFR2 and CD34 expression (Fig. 10D, E).

### Treatment response prediction of PHF5A in HCC

**Fig. 11:**
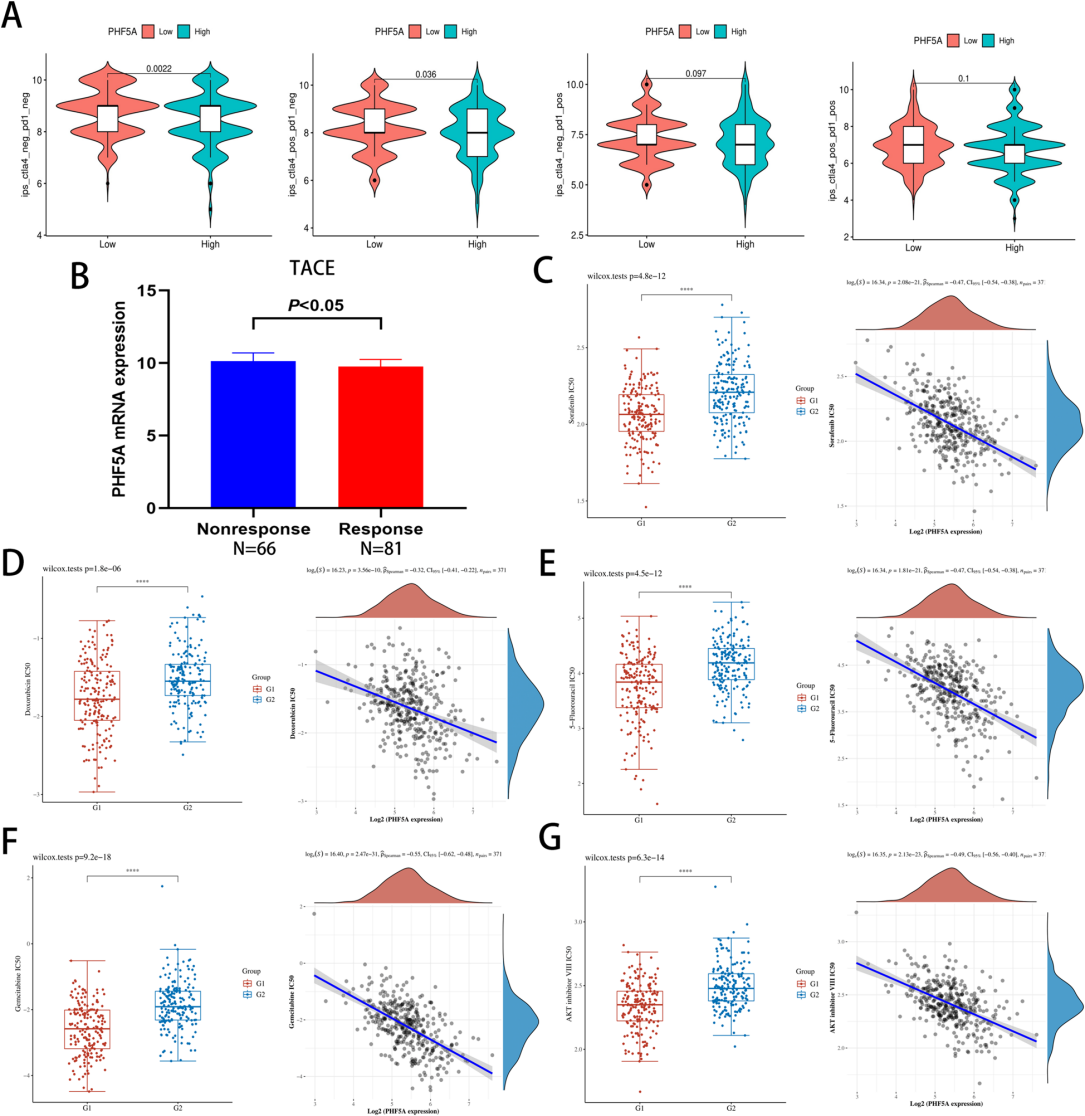
Correlation of PHF5A expression with treatment response in HCC. **A** Difference in immunotherapy efficacy of different PHF5A expression groups. **B** PHF5A expression in the nonresponsive and responsive groups to TACE. **C**-**G** Therapeutic effect of sorafenib, doxorubicin, 5-fluorouracil, gemcitabine and AKT inhibitor in different PHF5A expression groups. G1: high PHF5A expression group; G2: low PHF5A expression group

Given the impact of PHF5A on immune activities revealed across cancers analysis, its potential to forecast the outcome in terms of treatment benefits to immunotherapy was finally explored. For HCC patients with negative PD1 expression who received immunotherapy, the IPS was higher in low PHF5A patients than in high PHF5A patients, indicating that immunotherapy worked better in low PHF5A patients (Fig. 11A). Figure 11B indicated that low PHF5A patients performed better to TACE than high PHF5A patients. Additionally, other drugs (sorafenib, doxorubicin, 5-fluorouracil, gemcitabine and AKT inhibitor) were also analyzed. As shown in Fig. 11C-G, the IC50 values of these five drugs were lower in high PHF5A patients than in low PHF5A patients, indicating a higher sensitivity to the above drugs in patients with high PHF5A expression.

## Discussion

This study depicted the role of PHF5A across cancers from several public databases, which was followed by validation in HCC from the viewpoints of mRNA/protein expression, prognosis, immune infiltration, pathway enrichment, angiogenesis and treatment response. Bioinformatics analyses and validation experiments suggested that PHF5A was upregulated and predicted an unfavorable prognosis in various cancers and was confirmed to be associated with malignant phenotypes, unfavorable outcome, angiogenesis, immune evasion and treatment efficacy in HCC with the study cohort.

Aberrant PHF5A function exhibits a close link to development of a couple of tumors, as evidenced by accumulating studies [7–16]. Evidences including findings from our team have unveiled that PHF5A is significantly upregulated in NSCLC and enhances important malignant biological behaviors of cells via regulation of target genes and signaling pathways [8–10]. Liu et al. [16] discovered that PHF5A is markedly overexpressed in cells and tissues of head and neck squamous cell carcinoma (HNSCC), and PHF5A expression is correlated with primary tumor sites, T classifications, and clinical stages, and promotes HNSCC progression through p38 MAPK activation. For HCC, a previous study illustrated that PHF5A is highly expressed, and facilitates invasion and migration via the NF-KB pathway [19]. In line with these above previous studies, this study discovered that PHF5A expression markedly varied in tumor and matching normal tissues and was linked with functional status of diverse cancers at the single-cell level. PHF5A was revealed to participate in a couple of critical cell events in cancer, indicating that PHF5A may modulate tumor progression via diverse mechanisms. For example, PHF5A expression was correlated with the cell cycle in NSCLC in the study, and this result was supported by findings from previous studies that explored PHF5A’s effect on cell cycle in NSCLC [9, 38]. Moreover, PHF5A was demonstrated to be a prognostic biomarker by pan-cancer analysis. These results of this study corroborated with the previous findings by showing that highly PHF5A expression is associated with either poorer clinical stage of tumor or shorter survival time of patients in NSCLC [8, 9], breast cancer [11] or colorectal cancer [12, 13]. Mechanically, TMB and MSI can impact patients’ prognosis [39, 40]. TMB and MSI status have been identified to be tightly linked with PHF5A expression, indicating that PHF5A may affect patients’ survival by influencing TMB and MSI status.

The TME is formed depending on the communication of tumor cells with host cells [41]. The critical involvement of the TME in tumor development is increasingly being shown by research [42, 43]. This study revealed that PHF5A was highly linked to the TME and immune cell infiltration in many cancers. Ample immune cells that reside in the stroma around the nest of tumor cells without accessing these tumor cells have been proven to be a characteristic of the immune-exclusive tumor phenotype [44]. This study proved that PHF5A expression could affect the distribution of CD3+, CD4+, and CD8 + T cells in HCC. Hence, we speculated that PHF5A might have a link with tumor immune evasion. It is generally accepted that an elevated TIDE score indicates inferior performance of immune checkpoint blockade (ICB) and shorter survival after undergoing ICB treatment [45]. This study revealed that samples with elevated PHF5A expression had high TIDE scores in the majority of malignancies, indicating that patients with elevated PHF5A expression were more prone to tumor immune evasion, which is in line with our subsequent findings that in samples that were positive for PHF5A expression, CD3+, CD4+, and CD8 + T cells were concentrated in the tumor periphery and rarely infiltrated the tumor parenchyma. The findings that PHF5A may participate in tumor immune evasion were concurrently supported by the finding that immunotherapy worked better for HCC patients with low PHF5A expression in the validation study. Importantly, PHF5A expression was linked with key immune checkpoint genes, implying that PHF5A may contribute to the selection of immune checkpoint inhibitors and thus aid in clinical decision making.

To explore the potential oncogenic mechanisms of PHF5A in cancers, especially HCC, this study mainly focused on the exploration of signaling pathways activated by elevated PHF5A expression in HCC. GSEA revealed that PHF5A was involved in multiple signaling pathways, especially the Wnt signaling pathway [46], Notch signaling pathway [47], MAPK signaling pathway [48], mTOR signaling pathway [49], TGF-β signaling pathway [50], JAK STAT signaling pathway [51] and gap junction [52]. These identified pathways are classical oncogenic pathways affecting the oncogenesis and development of cancers, including HCC. This study also revealed that PHF5A may be engaged in immune- and vascular-related pathways, such as the T-cell receptor signaling pathway and VEGF signaling pathway, which is consistent with the immune-related findings mentioned above. The generation of new blood vessels for growth facilitates HCC development, and VEGF plays an essential role in this process [53, 54]. Consistently, abnormally high levels of angiogenesis often indicate an unfavorable prognosis, and advanced HCC patients treated with the anti-vascular drug sorafenib had significantly improved survival [55]. This study verified that PHF5A expression had a favorable connection with angiogenesis-related factors by applying bioinformatics and IHC staining, and we thus speculated that PHF5A may aid in HCC progression by enhancing angiogenesis, which is consistent with the finding that elevated PHF5A expression is linked to an adverse outcome in HCC patients. Furthermore, sorafenib, AKT inhibitor, and three commonly used chemotherapeutic drugs were more effective in patients who had high PHF5A expression. These outcomes support that PHF5A has a substantial effect on the angiogenic and proliferative state of HCC cells, and thus, patients with high PHF5A expression tend to exhibit an increased response to both antiangiogenic drugs and antiproliferative chemotherapeutic drugs. Most importantly, HCC patients with low PHF5A expression were observed to show a better response to TACE, suggesting that PHF5A is also helpful in selecting the optimal population for TACE therapy.

Combining the above findings, we could envision that for HCC patients with high PHF5A expression, they might have an intrinsic poor prognosis, and harbor tumor that prone to angiogenesis and immune evasion. Therefore, the treatment for these HCC patients should be strengthened, and follow-up should be more frequently. For optimal therapeutic options in these patients, we could consider that antiangiogenic drugs and antiproliferative chemotherapeutic drugs might work better than immunotherapy and TACE therapy.

## Conclusions

In summary, this study provides a new perspective for an enhanced comprehension of the value of PHF5A and its clinical application in human cancers. PHF5A has a critical function in diverse cancers and might be identified as an emerging prognostic marker and therapeutic target. Importantly, PHF5A has potential as a guiding biomarker for clinical prognosis evaluation and personalized treatment in HCC.

### Supplementary Information

Supplementary Material 1.

Supplementary Material 2.

Supplementary Material 3.

Supplementary Material 4.

Supplementary Material 5.

Supplementary Material 6.

Supplementary Material 7.

## Data Availability

The datasets used and/or analyzed during the current study available from the corresponding author on reasonable request.

## Abbreviations

ACLBI: Assistant for Clinical Bioinformatics
DSS: Disease-specific survival
GEO: Gene Expression Omnibus
GSEA: Gene set enrichment analysis
HCC: Hepatocellular carcinoma
IC50: The median inhibitory concentration
ICB: Immune checkpoint blockade
IHC: Immunohistochemistry
IPS: Immune phenotype score
MSI: Microsatellite instability
NES: Normalized enrichment score
OS: Overall survival
PFI: Progression-free interval
PHF5A: PHD-finger domain protein 5 A
TACE: Transcatheter arterial chemoembolization
TCGA: The Cancer Genome Atlas
TIDE: Tumor immune dysfunction and exclusion
TME: Tumor microenvironment
TMB: Tumor mutation burden
