# Supplementary figures and images for: Comprehensive analysis of PHF5A as a potential prognostic biomarker and therapeutic target across cancers and in hepatocellular carcinoma
Source: BMC Cancer. 2024 Jul 19;24:868. doi: 10.1186/s12885-024-12620-z (PMC11264801; doi:10.1186/s12885-024-12620-z)

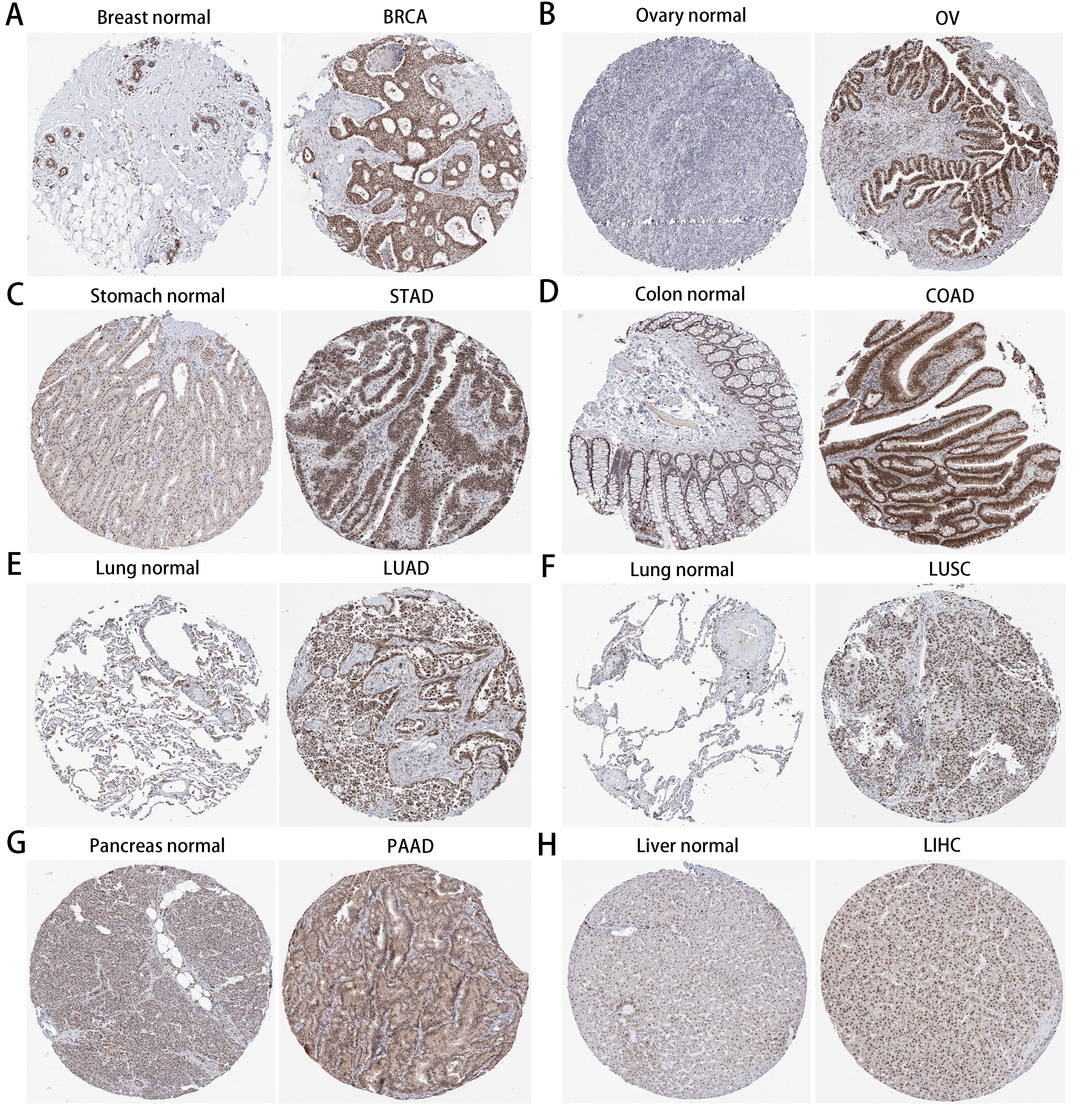

Supplement: Supplementary file 1 — Supplementary Material 1. [file 12885_2024_12620_MOESM1_ESM.tif]

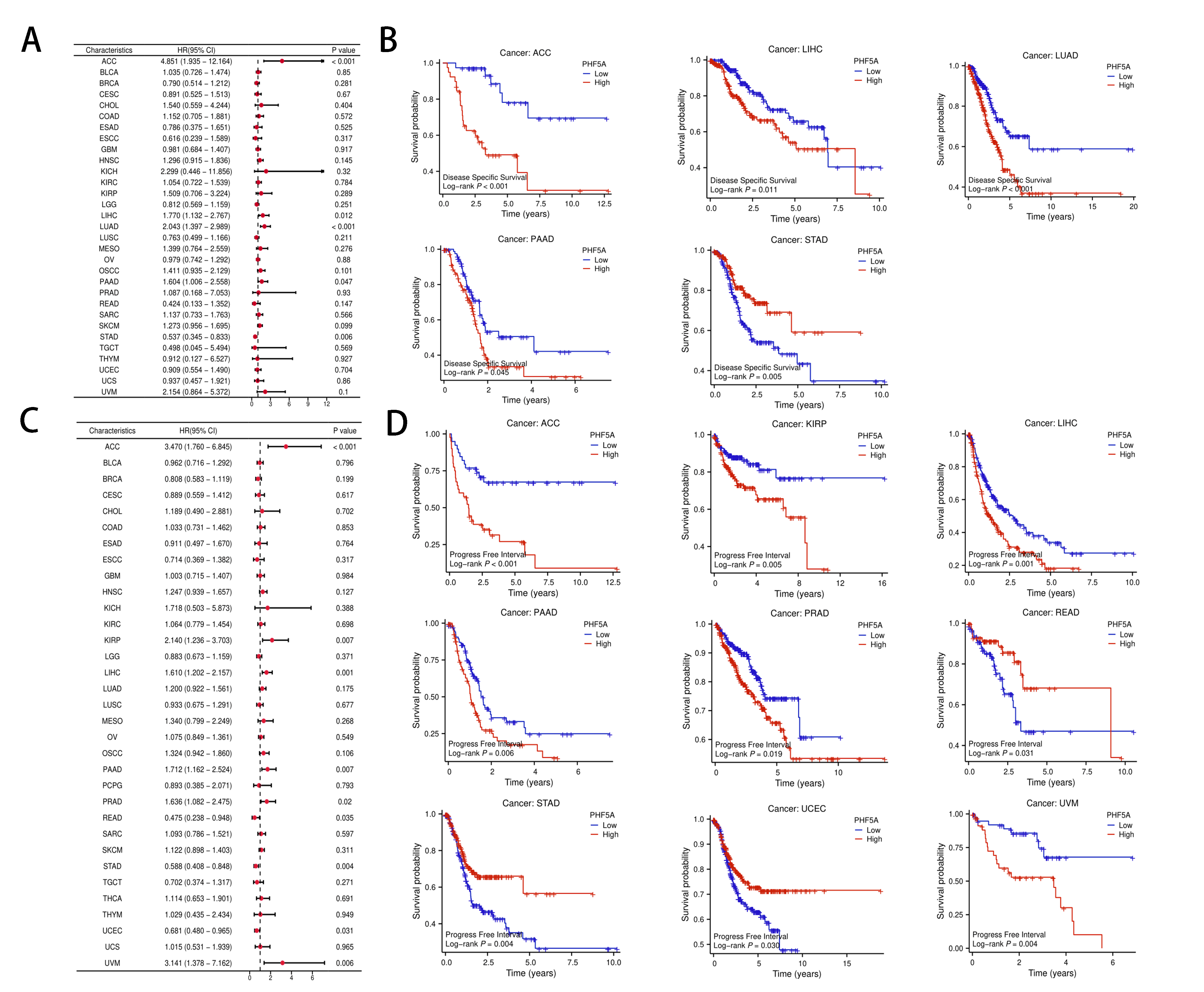

Supplement: Supplementary file 2 — Supplementary Material 2. [file 12885_2024_12620_MOESM2_ESM.tif]

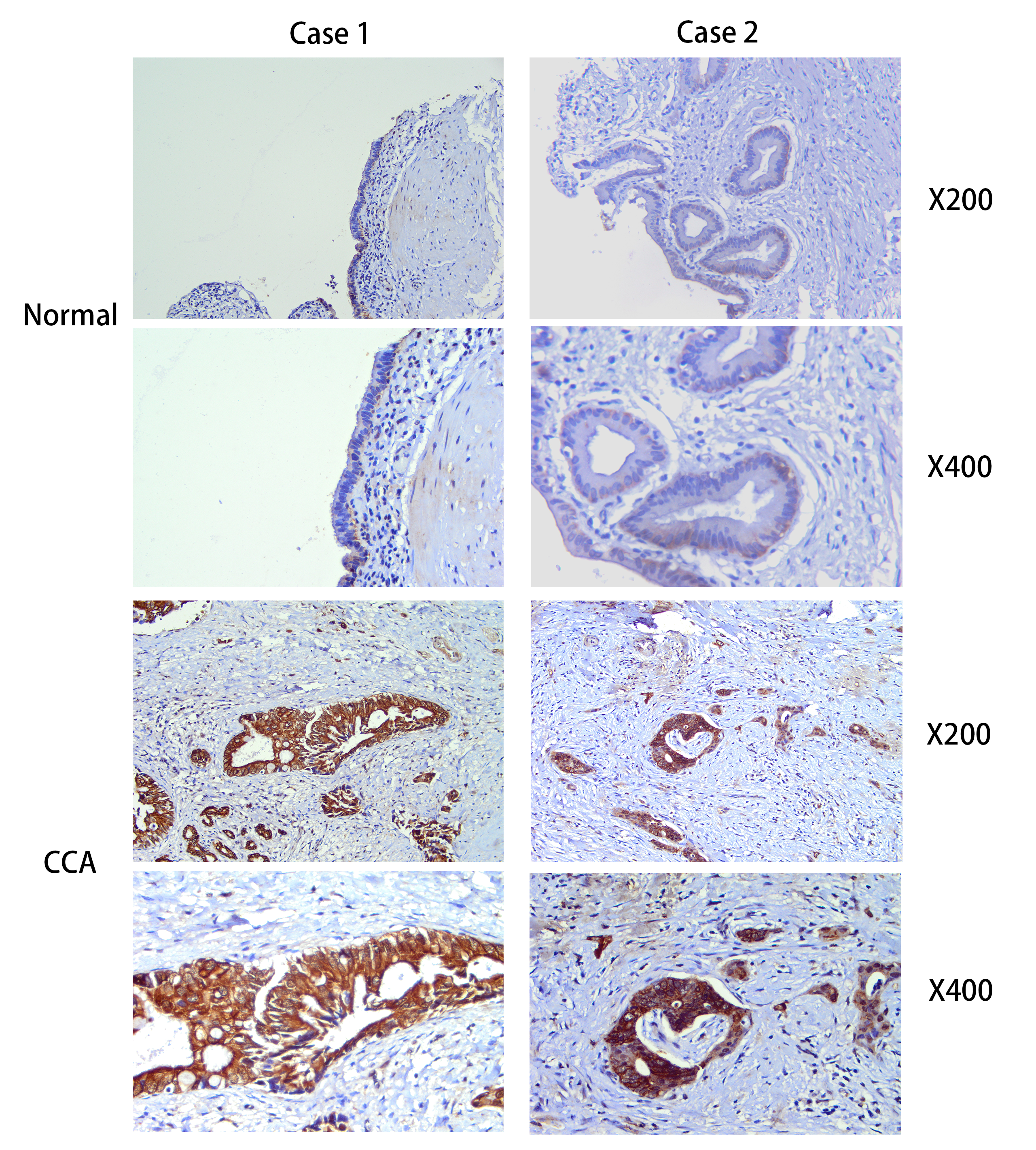

Supplement: Supplementary file 3 — Supplementary Material 3. [file 12885_2024_12620_MOESM3_ESM.tif]
