# Supplementary material for: Comprehensive analysis of PHF5A as a potential prognostic biomarker and therapeutic target across cancers and in hepatocellular carcinoma
Source: BMC Cancer. 2024 Jul 19;24:868. doi: 10.1186/s12885-024-12620-z (PMC11264801; doi:10.1186/s12885-024-12620-z)
Supplement: Supplementary file 4 — Supplementary Material 4. [file 12885_2024_12620_MOESM4_ESM.docx]

**Supplementary Table 1** The acronyms and matching full names of cancers

| **Cancer Type** | **Abbreviation** |
| --- | --- |
| adrenocortical carcinoma | ACC |
| bladder urothelial carcinoma | BLCA |
| breast invasive carcinoma | BRCA |
| cervical squamous cell carcinoma and endocervical adenocarcinoma | CESC |
| cholangiocarcinoma | CHOL |
| colon adenocarcinoma | COAD |
| lymphoid neoplasm diffuse large B-cell lymphoma | DLBC |
| esophageal carcinoma | ESCA |
| esophageal adenocarcinoma | ESAD |
| esophageal squamous cell carcinoma | ESCC |
| glioblastoma multiforme | GBM |
| head and neck squamous cell carcinoma | HNSC |
| kidney chromophobe | KICH |
| kidney renal clear cell carcinoma | KIRC |
| kidney renal papillary cell carcinoma | KIRP |
| acute myeloid leukemia | LAML |
| brain lower grade glioma | LGG |
| liver hepatocellular carcinoma | LIHC |
| non-small cell lung cancer | NSCLC |
| lung adenocarcinoma | LUAD |
| lung squamous cell carcinoma | LUSC |
| mesothelioma | MESO |
| ovarian serous cystadenocarcinoma | OV |
| oral squamous cell carcinoma | OSCC |
| pancreatic adenocarcinoma | PAAD |
| pheochromocytoma and paraganglioma | PCPG |
| prostate adenocarcinoma | PRAD |
| rectum adenocarcinoma | READ |
| sarcoma | SARC |
| skin cutaneous melanoma | SKCM |
| stomach adenocarcinoma | STAD |
| testicular germ cell tumors | TGCT |
| thyroid carcinoma | THCA |
| thymoma | THYM |
| uterine corpus endometrial carcinoma | UCEC |
| uterine carcinosarcoma | UCS |
| uveal melanoma | UVM |
