# Supplementary material for: Comprehensive analysis of PHF5A as a potential prognostic biomarker and therapeutic target across cancers and in hepatocellular carcinoma
Source: BMC Cancer. 2024 Jul 19;24:868. doi: 10.1186/s12885-024-12620-z (PMC11264801; doi:10.1186/s12885-024-12620-z)
Supplement: Supplementary file 5 — Supplementary Material 5. [file 12885_2024_12620_MOESM5_ESM.docx]

**Supplementary Table 2** Primers used for qRT‒PCR analysis

| Gene | Forward (5’-3’) | Reverse (5’-3’) | Product size (bps) |
| --- | --- | --- | --- |
| PHF5A | ACGGCTTCAAGAAGAGGTGA | TCCATCCCTACCACGTGTCT | 197 |
| GAPDH | TGAACGGGAAGCTCACTG | GCTTCACCACCTTCTTGATG | 120 |
