# Supplementary material for: Comprehensive analysis of PHF5A as a potential prognostic biomarker and therapeutic target across cancers and in hepatocellular carcinoma
Source: BMC Cancer. 2024 Jul 19;24:868. doi: 10.1186/s12885-024-12620-z (PMC11264801; doi:10.1186/s12885-024-12620-z)
Supplement: Supplementary file 6 — Supplementary Material 6. [file 12885_2024_12620_MOESM6_ESM.docx]

**Supplementary Table 3** Association between clinicopathological variables and PHF5A expression in HCC patients in the study cohort

| Factors | *N* | PHF5A-negative | PHF5A-positive | *χ^2^* | *P* |
| --- | --- | --- | --- | --- | --- |
| Gender |  |  |  |  |  |
| Female | 12 | 7(58.3%) | 5(41.7%) | Fisher | 0.732 |
| Male | 32 | 21(65.6%) | 11(34.4%) |  |  |
| Age (year) |  |  |  |  |  |
| ≤60 | 24 | 15(62.5%) | 9(37.5%) | 0.029 | 0.864 |
| >60 | 20 | 13(65.0%) | 7(35.0%) |  |  |
| Hepatitis |  |  |  |  |  |
| No | 7 | 4(57.1%) | 3(42.9%) | Fisher | 0.692 |
| Yes | 37 | 24(64.9%) | 13(35.1%) |  |  |
| AFP (ng/ml) |  |  |  |  |  |
| ≤20 | 22 | 17(77.3%) | 5(22.7%) | 3.536 | 0.060 |
| >20 | 22 | 11(50.0%) | 11(50.0%) |  |  |
| Child-Pugh |  |  |  |  |  |
| A | 40 | 26(65.0%) | 14(35.0%) | Fisher | 0.614 |
| B | 4 | 2(50.0%) | 2(50.0%) |  |  |
| Tumor size (cm) |  |  |  |  |  |
| ≤5 | 26 | 21(80.8%) | 5(19.2%) | 8.062 | 0.005* |
| >5 | 18 | 7(38.9%) | 11(61.1%) |  |  |
| Tumor number |  |  |  |  |  |
| 1 | 34 | 22(64.7%) | 12(35.3%) | Fisher | 1.000 |
| ≥2 | 10 | 6(60.0%) | 4(40.0%) |  |  |
| BCLC stage |  |  |  |  |  |
| A | 19 | 18(94.7%) | 1(5.3%) | 13.977 | 0.000* |
| B | 25 | 10(40.0%) | 15(60.0%) |  |  |
| Postoperative therapy |  |  |  |  |  |
| No | 15 | 10(66.7%) | 5(33.3%) | 0.090 | 0.764 |
| Yes | 29 | 18(62.1%) | 11(37.9%) |  |  |

**Notes:** **P*<0.05
