# Supplementary material for: Comprehensive analysis of PHF5A as a potential prognostic biomarker and therapeutic target across cancers and in hepatocellular carcinoma
Source: BMC Cancer. 2024 Jul 19;24:868. doi: 10.1186/s12885-024-12620-z (PMC11264801; doi:10.1186/s12885-024-12620-z)
Supplement: Supplementary file 7 — Supplementary Material 7. [file 12885_2024_12620_MOESM7_ESM.docx]

**Supplementary Table 4** GSEA pathways activated by up-regulation of PHF5A in HCC

| Gene sets | NES | NOM *p*-val | FDR q-val |
| --- | --- | --- | --- |
| KEGG_WNT_SIGNALING_PATHWAY | 1.93 | 0.000* | 0.003 |
| KEGG_NOTCH_SIGNALING_PATHWAY | 1.87 | 0.000* | 0.005 |
| KEGG_MAPK_SIGNALING_PATHWAY | 1.79 | 0.002* | 0.009 |
| KEGG_MTOR_SIGNALING_PATHWAY | 1.79 | 0.000* | 0.009 |
| KEGG_VEGF_SIGNALING_PATHWAY | 1.74 | 0.002* | 0.012 |
| KEGG_TGF_BETA_SIGNALING_PATHWAY | 1.70 | 0.013* | 0.018 |
| KEGG_JAK_STAT_SIGNALING_PATHWAY | 1.68 | 0.006* | 0.021 |
| KEGG_T_CELL_RECEPTOR_SIGNALING_PATHWAY | 1.65 | 0.012* | 0.025 |
| KEGG_GAP_JUNCTION | 1.60 | 0.016* | 0.038 |

**Notes:** **P*<0.05
